# Discriminatory Precision of Neutrophil Gelatinase-Associated Lipocalin in Detection of Urinary Tract Infection in Children: a Systematic Review and Meta-Analysis

**Published:** 2020-04-25

**Authors:** Arash Abbasi, Fardin Nabizadeh, Maryam Gardeh, Kosar Mohamed Ali, Mahmoud Yousefifard, Mostafa Hosseini

**Affiliations:** 1 Pediatric Chronic Kidney Disease Research Center, Tehran University of Medical Sciences, Tehran, Iran.; 2 Department of Pediatrics, Division of Nephrology, Children’s Medical Center, Tehran University of Medical Sciences, Tehran, Iran.; 3 Student Research Committee, Iran University of Medical Sciences, Tehran, Iran.; 4 College of Medicine, University of Sulaimani, Sulaimani, Iraq.; 5 Physiology Research Center, Iran University of Medical Sciences, Tehran, Iran.; 6 Department of Epidemiology and Biostatistics, School of Public Health, Tehran University of Medical Sciences, Tehran, Iran.

**Keywords:** Lipocalin-2, Urinary Tract Infections, Child, Predictive Value of Tests

## Abstract

**Introduction:**

There is a significant discrepancy between studies on diagnostic precision of neutrophil gelatinase-associated lipocalin (NGAL) in diagnosis of urinary tract infection (UTI). Therefore, the present systematic review and meta-analysis was designed to assess the diagnostic value of NGAL in diagnosis of UTI in children and adolescents.

**Methods:**

An extensive search was performed on Medline, Embase, Scopus and Web of Science databases by the end of 2019. Two independent researchers screened and summarized the data. Discriminatory precision of urinary and serum NGAL was assessed by reporting area under the curve, sensitivity, specificity and diagnostic odds ratio with 95% confidence interval (95% CI).

**Results:**

Data from 12 studies were included. The area under the curve of urinary and serum NGAL for diagnosis of UTI in children and adolescents at the best cut-off point (between 30-39.9 ng/ml) was 0.95 (95% CI: 0.93 to 0.97) and 0.83 (95% CI: 0.80 to 0.86), respectively. Sensitivity, specificity and diagnostic odds ratio on urinary NGAL at these cut-off points were 0.89 (95% CI: 0.64 to 0.97), 0.89 (95% CI: 0.71 to 0.97) and 67 (95% CI: 5 to 891), respectively. Sensitivity, specificity and diagnostic odds ratio of serum NGAL in UTI detection were 0.85 (95% CI: 0.70 to 0.90), 0.81 (95% CI: 0.69 to 0.88) and 9.53 (95% CI: 1.52 to 59.65), respectively.

**Conclusion:**

The present meta-analysis showed that urinary NGAL had a high diagnostic value in detection of UTI in children and adolescents with an optimum cut-off point in the range of 30-39.9 ng/ml.

## Introduction

Urinary tract infection (UTI) is one of the most common childhood diseases, with a prevalence rate of 30% (1, 2). Data presented in 2007 showed that there were 10.5 million visits due to UTI (3), most of which occurred within one year after the first episode of UTI (4). This disease leads to serious complications such as renal scar, renal failure and hypertension (5, 6). Although UTI is a common condition among children, the nonspecific symptoms of UTI in this age group makes urine culture the sole reliable diagnostic test. However, the high chance of contamination of the sample and the relatively long waiting time for the results of the culture test, may postpone the diagnosis of UTI in children.

Some studies suggest that a combination of ultrasound, scintigraphy, and voiding cystography could improve the diagnosis of UTI (7, 8). However, even these imaging techniques do not have an acceptable diagnostic value in detecting UTI. 

In the meantime, use of urinary biomarkers may be helpful in diagnosis of UTI. Neutrophil gelatinase-associated lipocalin (NGAL) has been introduced as a diagnostic biomarker for kidney disease in numerous studies. For example, in two previous meta-analyses, we found that serum/urinary concentration of NGAL has an acceptable value in diagnosis of acute kidney injury (9, 10). There are also other studies that have evaluated the value of NGAL in identifying UTI. For example, Valdimarsson et al. in 2017 showed that urinary NGAL has a sensitivity and specificity of 96 to 100% in diagnosis of UTI (11). Similar findings have been reported by Lee et al. (12). But Kitao et al. suggested that urinary NGAL level in patients with UTI has low precision in predicting patient outcomes (13). This disagreement led us to design the present systematic review and meta-analysis to determine the diagnostic value of NGAL in detection of UTI in children and adolescents.

## Methods

**- Study design**

The Meta-analysis of observational studies in epidemiology (MOOSE) guideline was used to summarize the evidence on value of NGAL in diagnosis of UTI in children and adolescents (14). We used a similar method with our previous published works to conduct the present meta-analysis (15-20).

**- Eligibility criteria**

In this systematic review and meta-analysis, all observational studies aimed at investigating the value of NGAL in detection of UTI in children and adolescents were included. Exclusion criteria were studies without a UTI group, duplicate articles, recurrent UTI, in vitro/animal studies, adult studies, and reviews.

PICO in the present study was defined as follows. Problem or study population (P): children with UTI; index test (I): urine and serum levels of NGAL; comparison (C): with non-UTI subjects; outcome (O): The desired outcome was the diagnostic value of NGAL in diagnosis of UTI.

- **Search strategy**

The databases searched for in the present study included Medline through the PubMed, Embase, Scopus and ISI Web of Science. This study examined all indexed studies from the establishment of the database to the end of 2019. In addition, we performed manual searches on Google search engine, Google Scholar and bibliography of related articles. Keywords associated with UTI in combination with words related to NGAL were used to perform the search in the present meta-analysis. The search strategy at Embase database is reported in appendix 1.

- **Data extraction**

The data were screened and summarized by two independent researchers. A checklist was used to summarize the data, including first author's name, year of publication, number of patients, age, sex, number of UTI and non-UTI patients, urinary and serum levels of NGAL, and follow-up duration. In addition, mean and standard deviation of NGAL level, NGAL cut-offs, true positive (TP), false positive (FP), true negative (TN), false negative (FN), sensitivity, specificity and area under the curve of NGAL were also recorded. The checklist was designed according to the PRISMA guidelines (21). It should be noted that after summarizing the data, the two researchers' checklists were compared and any disagreement was resolved by consulting a third researcher.

**- Quality control of articles**

Risk of bias among included studies was assessed based on quality assessment of diagnostic accuracy studies (QUADAS-2) recommendations (22). Disagreement was resolved through discussion with a third researcher.

**- Statistical analysis**

Analyses were performed using STATA 14.0 statistical software, applying the “midas” or “metan” commands. Analyses were stratified based on urinary and serum level of NGAL.

At first, mean and standard deviation of urinary and serum levels of NGAL were compared between UTI and non-UTI groups. Finding was presented as standardized mean difference (SMD) with 95% confidence interval (95% CI). Publication bias was assessed using Egger's test and presenting funnel plot.

In the second part, we evaluated the discriminatory power of NGAL in identifying UTI in children and adolescents. For this purpose, the data were summarized as TP, FP, TN and FN. Findings were reported as area under the summary receiver operating characteristics (SROC) curve (AUC), sensitivity, specificity and diagnostic odds ratio with 95% CI. Publication bias was examined in this section using Deek's funnel asymmetry test. In all analyses, heterogeneity was assessed using I^2^ test.

## Results

**Study design**

**Figure 1:**
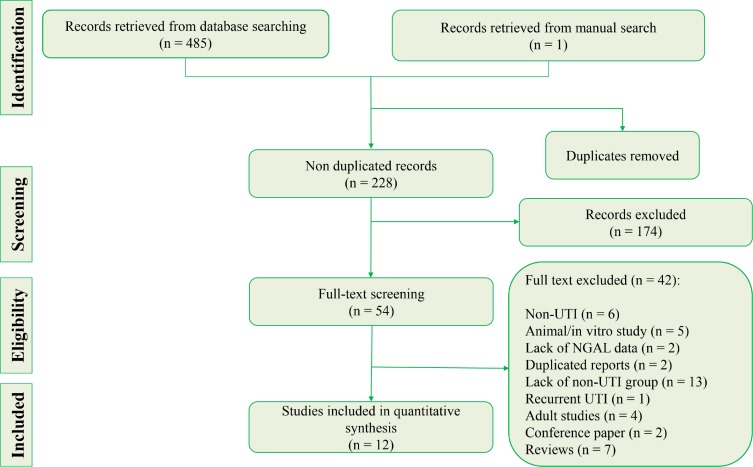
PRISMA flow diagram for the selection process

Database search eventually identified 485 articles. After eliminating duplicates and performing initial screening, 54 articles were studied in full. Of these, 42 articles were excluded. Reasons for excluding the papers included non-UTI studies (n = 6), animal/in vitro studies (n = 5), lack of NGAL data (n = 2), duplicated reports (n = 2), absence of non-UTI group (n = 13), recurrent UTI (n = 1), adult studies (n = 4), and review articles (n = 7). Two conference papers that did not present our required data were excluded. Before excluding these two papers, we tried to obtain the required data using two strategies. First, we performed an extensive database search to find other published articles of the authors. Second, we sent two repetitive emails to corresponding authors. Finally, data from 12 studies were included in the present meta-analysis (11, 23-33) (Figure 1). There were 5 prospective cross-sectional studies, 2 retrospective cross-sectional studies, and 5 case-control studies. The sample size of included studied varied from 62 patients to 444 patients (total sample size of 2112 children, 50.9% male). 724 children had UTI and 1388 were non-UTI. The most common method of measuring NGAL was ELISA. Nine studies assessed urinary NGAL, 1 study was performed on serum level of NGAL and 2 studies examined both serum and urine NGAL levels. Tables 1 and 2 present the characteristics of included studies.

**Risk of bias and publication bias**

**Figure 2:**
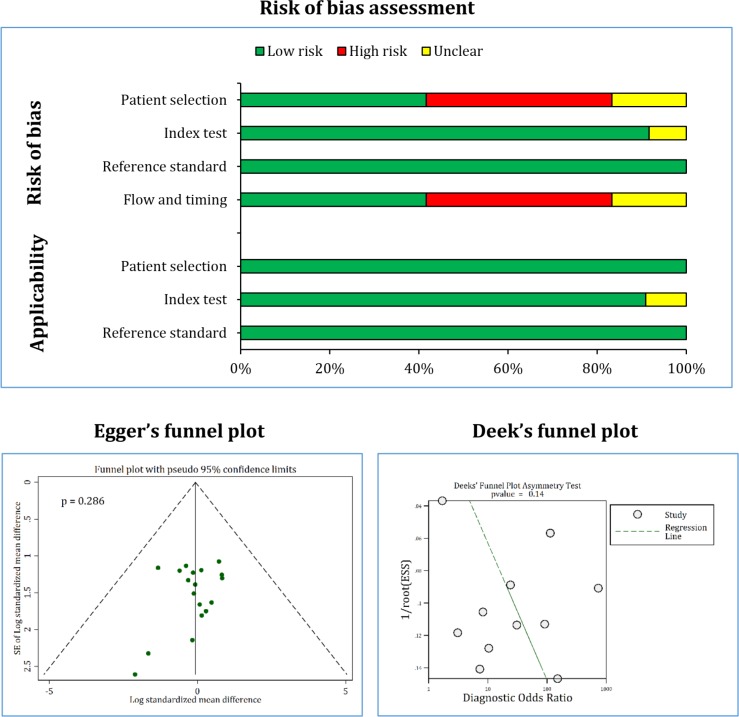
Risk of bias and publication bias among eligible studies. Risk of bias regarding patient selection and flow and timing was high in 5 studies and unclear in 2 studies. Risk of bias and applicability of index test was unclear in 1 study. There was no evidence of publication bias in standardized mean difference (p_Egger’s test _=0.286) and discriminatory accuracy (p_Deek’s test_=0.140) assessment of neutrophil gelatinase-associated lipocalin in detection of urinary tract infection

Quality control of the articles based on QUADAS-2 tools showed that the risk of bias in patient selection, flow and timing was high in 5 studies. This was due to the case-control nature of these studies. Sampling method was also retrospective in two studies. Therefore, the risk of bias in patient selection and flow and timing in these studies was unclear. In addition, the risk of bias and applicability of the index test was unclear in a study (Table 3, Figure 2). Finally, there was no evidence of publication bias in the present meta-analysis (Figure 2).

**Meta-analysis**

**- The mean difference of urinary and serum NGAL levels between the UTI and non-UTI patients**

**Figure 3:**
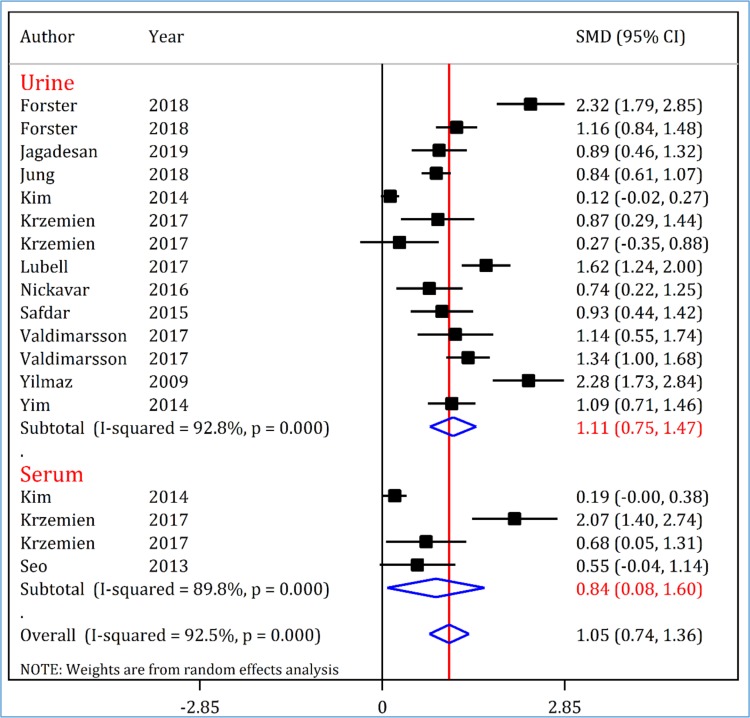
Standardized mean difference (SMD) of neutrophil gelatinase-associated lipocalin (NGAL) between urinary tract infected (UTI) and non-UTI children. Urine and serum levels of NGAL were significantly higher in UTI children. CI: Confidence interval

Figure 3 shows the mean difference of urinary and serum NGAL levels between UTI and non-UTI groups. As can be seen, both urinary levels (SMD = 1.11; 95% CI: 0.75 to 1.47; p <0.001; I^2^ = 92.8; p <0.001) and serum levels (SMD = 0.84; 95% CI: 0.08 to 1.60; p = 0.031; I^2^ = 89.8; p <0.001) of NGAL were significantly higher in UTI group compared to non-UTI group. As can be seen, there is high heterogeneity between studies. A subgroup analysis was performed to find the source of heterogeneity. We could not find the source of heterogeneity. Study design (p = 0.781), setting of patients (p = 0.147), sample size (p = 0.457), prevalence of UTI (p = 0.481) and NGAL assay method (p = 0.538) did not affect the SMD of urinary NGAL.

- **Diagnostic accuracy of urinary NGAL in detection of UTI**

**Figure 4:**
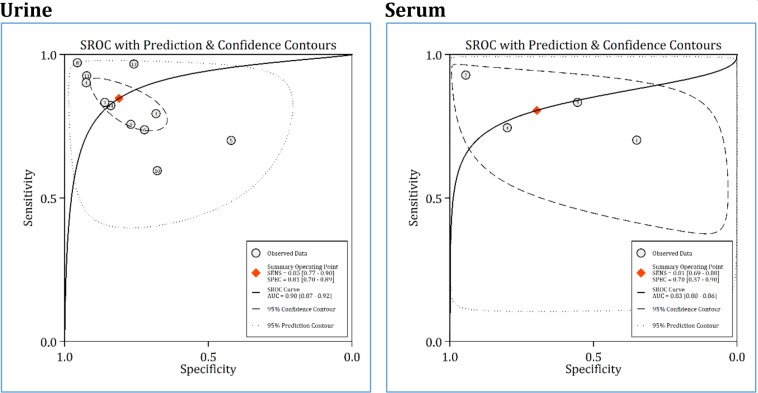
Area under the summary receiver operating characteristics (SROC) curve (AUC) of urine and serum neutrophil gelatinase-associated lipocalin (NGAL) in detection of urinary tract infection. CI: Confidence interval

**Figure 5:**
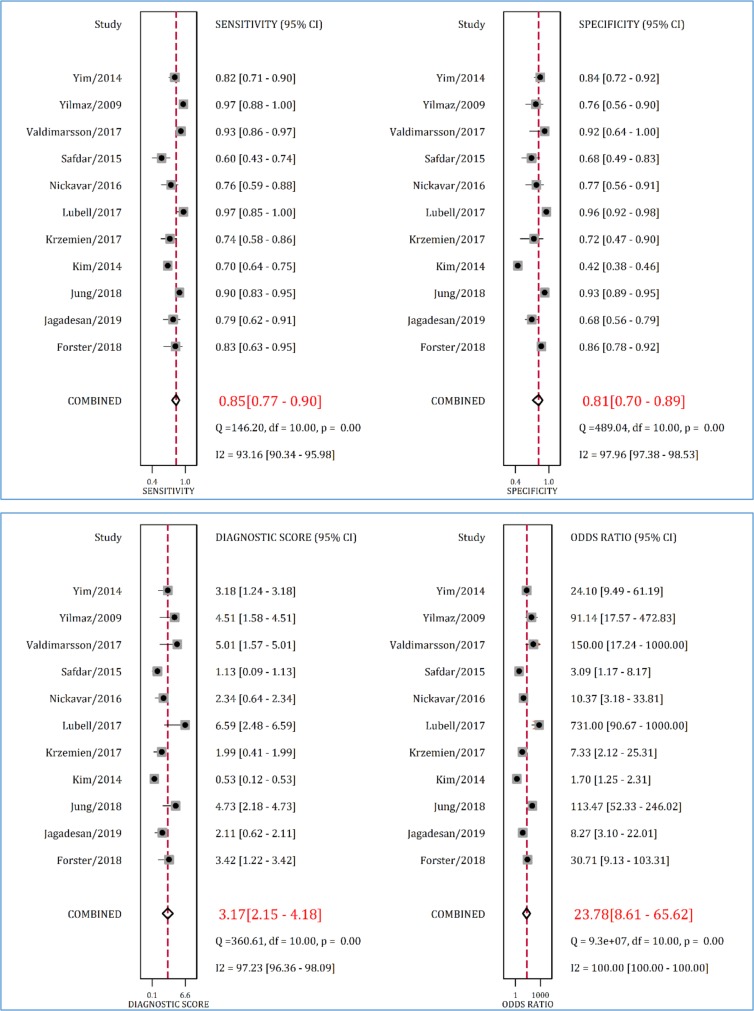
Sensitivity, specificity, diagnostic score and diagnostic odds ratio of urine neutrophil gelatinase-associated lipocalin (NGAL) in detection of urinary tract infection. CI: Confidence interval

Data from 11 studies were included in analyses of this section (11, 23-30, 32, 33). The AUC of urinary NGAL in diagnosis of UTI in children and adolescents was 0.90 (95% CI: 0.87 to 0.92) (Figure 4). Sensitivity and specificity of urinary NGAL in detecting UTI were 0.85 (95% CI: 0.77 to 0.90) and 0.81 (95% CI: 0.70 to 0.89), respectively (Figure 5). Additionally, diagnostic odds ratio of this urine biomarker was 23.78 (95% CI: 8.61 to 65.62).

Significant heterogeneity was also observed in this section (I^2^ between 93.16 to 100%). A subgroup analysis based on NGAL cut-off point was performed to find the source of heterogeneity. As Table 4 shows, the cut-off points reported in the studies were divided into four groups of urinary NGAL level <20 ng / ml, 20-29.9 ng / ml, 30-39.9 ng / ml and 40 ng / ml and over. We observed optimum performance of urinary NGAL in cut-off points of 30 to 39.9 ng / ml. Analyses showed that AUC of urinary NGAL in detection of UTI was 0.95 (95% CI: 0.93 to 0.97) when the cut-off point was in the range of 30-39.9 ng / ml. The sensitivity, specificity and diagnostic odds ratio of NGAL at these cut-off points were 0.89 (95% CI: 0.64 to 0.97), 0.89 (95% CI: 0.71 to 0.97) and 67 (95% CI: 5 to 891), respectively.

- **Diagnostic accuracy of serum NGAL in detection of UTI**

**Figure 6:**
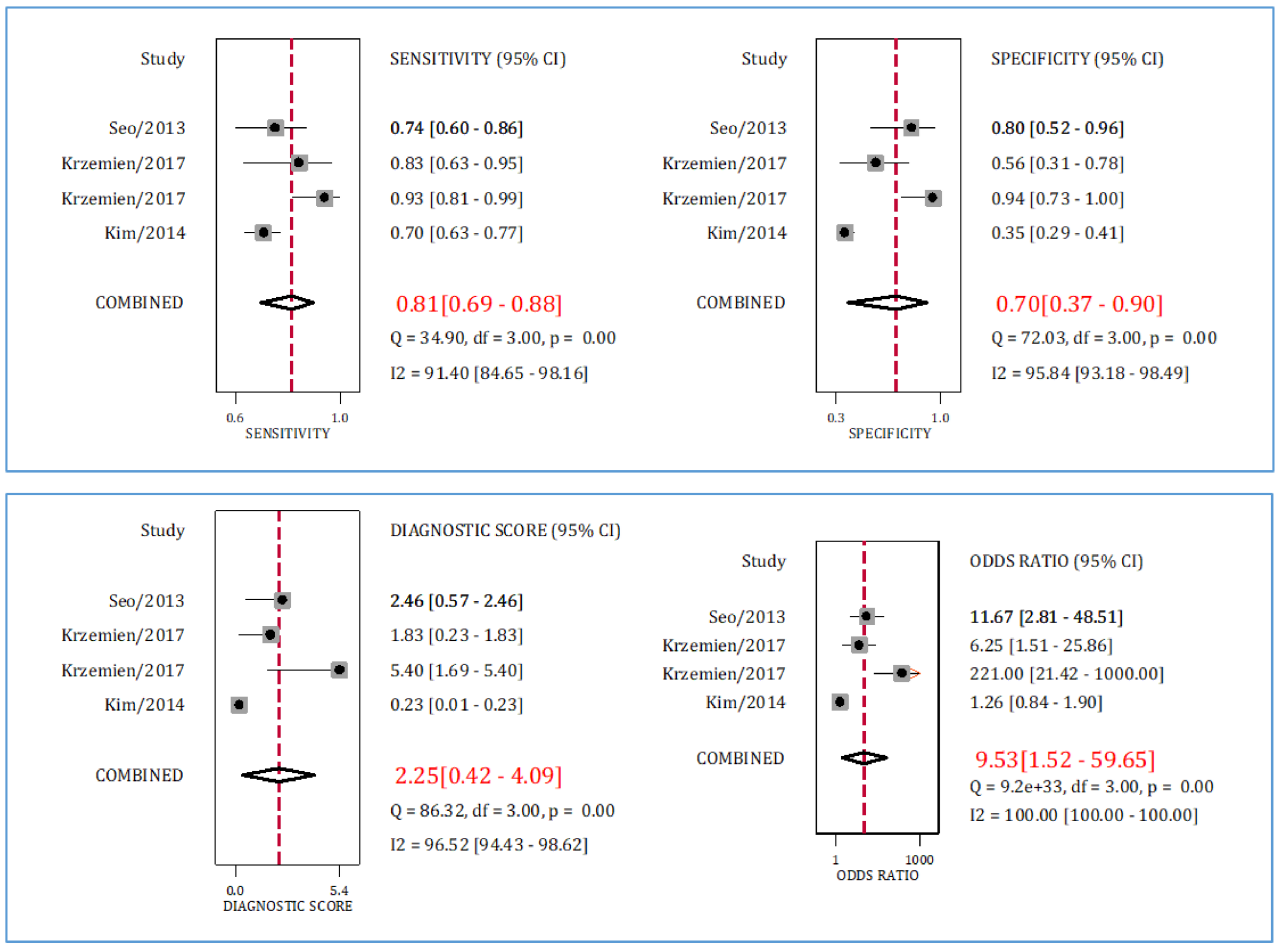
Sensitivity, specificity, diagnostic score and diagnostic odds ratio of serum neutrophil gelatinase-associated lipocalin (NGAL) in detection of urinary tract infection. CI: Confidence interval

Data from three studies were included in the analyses of this section (26, 27, 31). The AUC of serum NGAL in diagnosis of UTI in children and adolescents was 0.83 (95% CI: 0.80 to 0.86) (Figure 4). Sensitivity and specificity of serum NGAL in detecting UTI were 0.85 (95% CI: 0.70 to 0.90) and 0.81 (95% CI: 0.69 to 0.88), respectively (Figure 6). Diagnostic odds ratio of this serum biomarker was 9.53 (95% CI: 1.52 to 59.65). Despite the obvious heterogeneity, subgroup analysis was not performed because the number of articles was small.

## Discussion

The present meta-analysis showed that NGAL is able to detect UTI in children and adolescents. Stratified analysis showed that the urinary level of this biomarker had higher discriminatory power than its serum level and the optimum cut-off point was in the range of 30-39.9 ng / ml. When we assess the discriminatory precision of a test/biomarker, diagnostic odds ratio indicates the usefulness of the test/biomarker in the clinic. Urinary NGAL at cut-off points between 30-39.9 ng / ml had a diagnostic odds ratio of 67, which indicated its high applicability in diagnosis of UTI in children.

In the present study, significant heterogeneity was observed among the studies. Unfortunately, the origin of this heterogeneity was not determined by subgroup analysis. The only factor that partially reduced the heterogeneity was the difference in cut-off points. It should be noted that the concept of heterogeneity has changed in recent years. In general, tests used for heterogeneity are low powered so that they may not be able to detect heterogeneity when the number of articles is small. On the other hand, the test may falsely show a heterogeneity when the number of included articles was high. For this reason, researchers have recently suggested that numerical values of heterogeneity should be reported as descriptive statistics and it should be interpreted intuitively from the clinical perspective (34). The sensitivity and specificity reported in most studies included in the present meta-analysis were above 70%. Clinically, this level of sensitivity and specificity is divided into good and excellent groups, both of which represent good discriminatory performance of NGAL in diagnosis of UTI in children. Although the diversity of reported sensitivity and specificity between the studies was not clinically high, the I^2^ test indicated high heterogeneity between eligible studies. Therefore, it seems that the heterogeneity reported in I^2^ test has no clinically significant effect on the reported results.

The aim of the present study was to evaluate the value of NGAL in the first episode of UTI in children and adolescents. Therefore, the diagnostic value of NGAL in recurrent UTI was excluded. The reason for these exclusion criteria was reducing heterogeneity between studies. The pattern of urinary NGAL levels in recurrent UTI appears to be different from the first episode of UTI. For example, in 2017, Forster et al. showed that urinary NGAL level in recurrent UTI patients was significantly lower than first episode UTI patients. These researchers suggest that a defect in urinary NGAL production may be a prerequisite for recurrent UTI (35). Of course, this is still a hypothesis and further studies are needed to reach a definite conclusion. 

Risk of bias regarding patient selection and flow and timing was high in 5 studies and unclear in 2 studies. This was due to the case-control design in 5 studies and retrospective sampling method in 2 other studies. However, since NGAL level was evaluated using a standard ELISA method, the applicability of the index test was rated as desirable in the present systematic review.

One of the issues that should be considered when examining diagnostic performance of a biomarker is its cost-effectiveness. Although NGAL may improve the management of children with UTI, its economic impact should also be considered. To the best of our knowledge, there is no study on the cost-effectiveness of NGAL in diagnosis of UTI in children. But Shaw et al. in 2011 showed that NGAL is a cost-effective biomarker for diagnosis of acute kidney injury (36). In another study involving 10,000 patients, Parikh et al. showed that use of urinary NGAL plus serum creatinine in diagnosis of acute kidney injury not only improves patient management but also reduces treatment costs (37).

**Table 1:** Characteristics of included studies in assessment of mean difference of NGAL in detection of urinary tract infection

**Author; year; country**	**Study type**	**Setting**	**Age**	**Sample size**	**No. of boys**	**number of patients UTI / Non UTI**	**UTI group**	**Control group**	**NGAL assay methods**	**Source of NGAL**
Forster; 2018; USA	PCS	Neurogenic bladder	<18	201	102	24 / 177	UTI	No UTI	ELISA	Urine
Jagadesan; 2019; India	PCS	Suspected UTI	0.2 to 5 years	100	43	34 / 66	UTI	No UTI	Turbidometric immunoassay	Urine
Jung; 2018; Korea	RCS	Suspected UTI	<3 month	422	267	102 / 320	UTI	No UTI	Microparticle immunoassays	Urine
Kim; 2014; Korea	RCS	Suspected UTI	3.87±2.59 years	444	177	107 / 337	UTI	No UTI	ELISA	Urine/Serum
Krzemien; 2017; Poland	PCS	Suspected UTI	1 to 12 months	84	42	66 / 18	Febrile and non-febrile UTI	No UTI	ELISA	Urine/Serum
Lubell; 2017; USA	PCS	Suspected UTI	0 to 2 years	260	118	35 / 225	Febrile UTI	Non-UTI	ELISA	Urine
Nickavar; 2016; Iran	CCS	UTI and non-UTI	0 to 12 years	63	23	37 / 26	UTI	Healthy control	ELISA	Urine
Safdar; 2015; Saudi Arabia	PCS	Suspected UTI	0 to 14 years	73	33	31 / 42	UTI	Non-UTI	NR	Urine
Seo; 2013; Korea	CCS	UTI and non-UTI	0-1 years	62	40	47 / 15	UTI	Healthy control	ELISA	Serum
Valdimarsson; 2017; Sweden	CCS	UTI and non-UTI	0-1 years	185	105	108 / 77	UTI	Healthy control	ELISA	Urine
Yilmaz; 2009; Turkey	CCS	UTI and non-UTI	0.1 to 14 years	89	24	60 / 29	UTI	Healthy control	ELISA	Urine
Yim; 2014; Korea	CCS	UTI and non-UTI	Mean 23 months	129	84	73 / 56	UTI	Healthy control	Immunosorbent assay	Urine

CCS: Case-Control study; ELISA: enzyme-linked immunosorbent assay; NGAL: neutrophil gelatinase-associated lipocalin; PCS: Prospective cross-sectional study; RCS: Cross-sectional study; UTI: Urinary tract infection.

**Table 2:** Characteristics of included studies in assessment of diagnostic accuracy of NGAL in detection of urinary tract infection

Author; year; country	**Sample size**	**No. of boys**	**number of patients UTI / Non UTI**	**UTI group**	**Control group**	**Source of NGAL**	**Cut-off ng/ml**	**TP**	**FP**	**FN**	**TN**
Forster; 2018; USA	201	102	24 / 100	UTI	No UTI	Urine	229	20	14	4	86
Jagadesan; 2019; India	100	43	34 / 66	UTI	No UTI	Urine	27	27	21	7	45
Jung; 2018; Korea	422	267	102 / 320	UTI	No UTI	Urine	46.2	92	24	10	296
Kim; 2014; Korea	444	177	107 / 337	UTI	No UTI	Urine	5.75	199	306	85	222
						Serum	65.35	134	151	57	81
Krzemien; 2017; Poland	84	42	42 / 18	Febrile UTI	No UTI	Urine	42.2	31	5	11	13
				Febrile UTI	No UTI	Serum	76.2	39	1	3	17
				Non-Febrile UTI	No UTI	Serum	39	20	8	4	10
Lubell; 2017; USA	260	118	35 / 225	Febrile UTI	Non-UTI	Urine	39.1	34	10	1	215
Nickavar; 2016; Iran	63	23	37 / 26	UTI	Healthy control	Urine	0.2	28	6	9	20
Safdar; 2015; Saudi Arabia	73	33	31 / 42	UTI	Non-UTI	Urine	35.83	25	10	17	21
Seo; 2013; Korea	62	40	47 / 15	UTI	Healthy control	Serum	61	35	3	12	12
Valdimarsson; 2017; Sweden	185	105	108 / 77	UTI	Healthy control	Urine	38	100	1	8	12
Yilmaz; 2009; Turkey	89	24	60 / 29	UTI	Healthy control	Urine	20	58	7	2	22
Yim; 2014; Korea	129	84	73 / 56	UTI	Healthy control	Urine	23.95	60	9	13	47

FN: False Negative; FP: False positive; NGAL: neutrophil gelatinase-associated lipocalin; UTI: Urinary tract infection; TN: True negative; TP: True positive.

**Table 3:** Risk of bias assessment among included studies

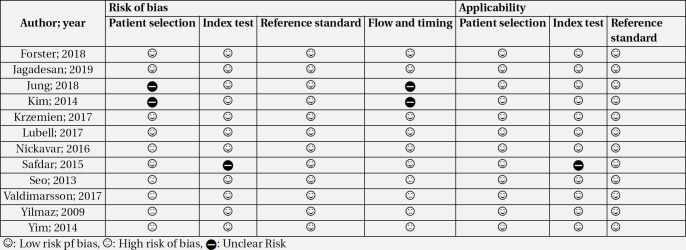

**Table 4:** Diagnostic performance of NGAL in detection of UTI at different cut-offs

Cut offs	**AUC**	**Sensitivity**	**Specificity**	**Positive LR**	**Negative LR**	**Odds ratio**
<20 ng/ml	0.73 (0.69 to 0.77)	0.73 (0.63 to 0.80)	0.58 (0.32 to 0.80)	1.7 (0.9 to 3.4)	0.47 (0.25 to 0.89)	4 (1 to 14)
20-29.9 ng/ml	0.85 (0.81 to 0.88)	0.88 (0.74 to 0.95)	0.76 (0.66 to 0.84)	3.7 (2.5 to 5.4)	0.16 (0.07 to 0.36)	23 (8 to 67)
30-39.9 ng/ml	0.95 (0.93 to 0.97)	0.89 (0.64 to 0.97)	0.89 (0.71 to 0.97)	8.3 (2.4 to 28.7)	0.12 (0.03 to 0.53)	67 (5 to 891)
>40 ng/ml	0.92 (0.89 to 0.94)	0.84 (0.72 to 0.91)	0.87 (0.79 to 0.93)	6.4 (3.2 to 12.8)	0.19 (0.10 to 0.35)	34 (10 to 117)

AUC: area under the curve; LR: likelihood ratio.

## Conclusion

The present meta-analysis showed that urinary NGAL had a high diagnostic value in detection of UTI in children and adolescents with an optimum cut-off point in the range of 30-39.9 ng/ml.
